# Dipeptidyl Peptidase Inhibition Enhances CD8 T Cell Recruitment and Activates Intrahepatic Inflammasome in a Murine Model of Hepatocellular Carcinoma

**DOI:** 10.3390/cancers13215495

**Published:** 2021-11-01

**Authors:** James M. Henderson, Michelle S. W. Xiang, Jiali Carrie Huang, Stefanie Wetzel, Linxuan Jiang, Jack H. Lai, Wengen Wu, James G. Kench, William W. Bachovchin, Ben Roediger, Geoffrey W. McCaughan, Hui Emma Zhang, Mark D. Gorrell

**Affiliations:** 1 Centenary Institute, Faculty of Medicine and Health, The University of Sydney, Camperdown, NSW 2006, Australia; jhen7621@gmail.com (J.M.H.); m.xiang@centenary.org.au (M.S.W.X.); jhua9515@uni.sydney.edu.au (J.C.H.); christian.stefanie@yahoo.com (S.W.); ljia7302@uni.sydney.edu.au (L.J.); ben.roediger@novartis.com (B.R.); g.mccaughan@centenary.org.au (G.W.M.); 2 Institute for Cardiovascular Prevention, Ludwig-Maximillians-Universität, D-80336 Munich, Germany; 3 Sackler School of Graduate Biomedical Science, Tufts University, Boston, MA 02111, USA; jack.lai@tufts.edu (J.H.L.); WenGen.Wu@tufts.edu (W.W.); william.bachovchin@tufts.edu (W.W.B.); 4 Tissue Pathology & Diagnostic Oncology, NSW Health Pathology, Royal Prince Alfred Hospital, Camperdown, NSW 2050, Australia; James.Kench@sydney.edu.au; 5 A.W. Morrow Gastroenterology and Liver Centre, Royal Prince Alfred Hospital, Camperdown, NSW 2050, Australia

**Keywords:** ARI-4175, pan-DPP inhibitor, DPP4, HCC, caspase-1

## Abstract

**Simple Summary:**

This study reported, for the first time, on the expression and activity of the dipeptidyl peptidase 4 (DPP4) family during the development of hepatocellular carcinoma (HCC). We also demonstrated that the pan-DPP inhibitory compound ARI-4175 significantly reduced the number of macroscopic liver nodules in a mouse HCC model. ARI-4175 increased intrahepatic inflammatory cell infiltration, CD8^+^ T cell numbers and caspase-1-mediated inflammasome activation in the HCC-bearing liver. Thus, this study provides promising data on the efficacy of ARI-4175 in the treatment of early-stage HCC. Targeting the DPP4 family may be a novel and effective approach to promote anti-tumour immunity in HCC via caspase-1 activation.

**Abstract:**

The mRNA expression of the dipeptidyl peptidase 4 (DPP4) gene family is highly upregulated in human hepatocellular carcinoma (HCC) and is associated with poor survival in HCC patients. Compounds that inhibit the DPP4 enzyme family, such as talabostat and ARI-4175, can mediate tumour regression by immune-mediated mechanisms that are believed to include NLRP1 activation. This study investigated the expression and activity of the DPP4 family during the development of HCC and evaluated the efficacy of ARI-4175 in the treatment of early HCC in mice. This first report on this enzyme family in HCC-bearing mice showed DPP9 upregulation in HCC, whereas intrahepatic DPP8/9 and DPP4 enzyme activity levels decreased with age. We demonstrated that ARI-4175 significantly lowered the total number of macroscopic liver nodules in these mice. In addition, ARI-4175 increased intrahepatic inflammatory cell infiltration, including CD8^+^ T cell numbers, into the HCC-bearing livers. Furthermore, ARI-4175 activated a critical component of the inflammasome pathway, caspase-1, in these HCC-bearing livers. This is the first evidence of caspase-1 activation by a pan-DPP inhibitor in the liver. Our data suggest that targeting the DPP4 enzyme family may be a novel and effective approach to promote anti-tumour immunity in HCC via caspase-1 activation.

## 1. Introduction

Liver cancer is the sixth most common cancer and the fourth most common cause of cancer mortality worldwide [1,2]. Hepatocellular carcinoma (HCC) is responsible for 70-85% of primary liver cancer cases [3].

Few therapeutic options exist for patients with HCC. Sorafenib and lenvatinib, multi-kinase inhibitors with anti-proliferative and anti-angiogenic effects, have shown only modest benefit in advanced and unresectable HCC [4]. In 2017, the US Food and Drug Administration approved nivolumab, an anti-programmed death-1 (PD-1) monoclonal antibody, to treat advanced HCC in patients who have been previously treated with sorafenib [5]. The evaluation of its clinical benefit is ongoing, although indications suggest a degree of benefit for 18% of patients for this immunomodulatory drug. More recently, combination therapy with atezolizumab (anti-PD-L1) and bevacizumab (anti-VEGF-A) has been associated with better outcomes, although some patients experienced significant side effects [6]. Thus, there is growing evidence of immunological control of HCC, but still an urgent unmet need to develop new cancer therapies.

The dipeptidyl peptidase 4 (DPP4) gene family includes four atypical serine proteases: DPP4, DPP8, DPP9 and fibroblast activation protein (FAP). These four peptidases mediate a diverse range of biological processes by hydrolysing the N-terminal dipeptide of substrates that have a proline or alanine at the penultimate position [7,8]. A comprehensive database interrogation has shown that mRNA expression levels of these four genes are highly upregulated in HCC tumours compared to non-diseased tissue [9]. Their joint high expression is associated with poor survival in HCC [9]. These findings emphasise the potential of the DPP4 enzyme family as a set of therapeutic targets in HCC.

Val-boroPro (also called PT-100, talabostat and BXCL701) is a first-generation pan-DPP inhibitory compound, which inhibits the proteolytic activity of the DPP4 enzyme family. Administration of Val-boroPro has been shown to mediate regression in multiple tumour models [10]. Val-boroPro can stimulate tumour immunity via the upregulation of cytokine and chemokine levels in tumour and lymphoid tissues [11]. Val-boroPro can also promote dendritic cell (DC) trafficking and accelerate CD4^+^ and CD8^+^ T cell priming and expansion towards tumour-associated antigens in mice [12].

ARI-4175 is a second-generation pan-DPP inhibitor, following Val-boroPro which can also mediate tumour regression through immune-mediated mechanisms. Oral administration of ARI-4175 can produce complete tumour regression and immunity in mice transplanted with bladder tumour cells or rhabdomyosarcoma cells [10]. ARI-4175 modulates the activity of myeloid DC and myeloid-derived suppressor cells (MDSCs), which can potentially contribute to improved tumour immunity [10]. ARI-4175 also enhances the activity of adoptive cellular therapy (ACT) and DC vaccination, resulting in the regression of established rhabdomyosarcoma, which is resistant to treatment with ACT or DC vaccine when either is used alone [10]. In addition, ARI-4175 treatment can induce marked regression of well-established solid lung tumours, both as a single agent and as an adjuvant to DC therapy and ACT [13].

The key targets of these pan-DPP inhibitory compounds are DPP4 and DPP9, which have important roles in immune regulation. In HCC, high DPP4 expression has been associated with poorer prognosis [14,15]. Inhibition of DPP4 activity increases the infiltration of natural killer (NK) and T cells into HCC tissue [14]. DPP4 inhibition blocks the cleavage of CXCL10 by DPP4, thereby increasing the chemotaxis of immune cells towards tumour tissue via the cognate receptor of CXCL10, CXCR3 [14,16]. DPP9 sequesters the C-terminus of NLRP1 and thereby acts as an endogenous inhibitor of NLRP1 [17,18]. DPP9 inhibition activates the NLRP1 inflammasome, leading to caspase-1 activation, secretion of cleaved interleukin-1β (IL-1β) and interleukin-18 (IL-18), and pyroptotic cell death [19,20]. Furthermore, Val-boroPro suppresses human acute myeloid leukemia (AML) growth in mouse models by triggering pyroptosis in AML cells via DPP9 inhibition [21]. These data point towards DPP4 and DPP9 as targets for immunotherapy in tumours.

The primary objective in this study was to determine whether ARI-4175 has efficacy in the treatment of early HCC in mice, and to investigate potential mechanisms.

## 2. Materials and Methods

### 2.1. Mouse Model and Experimental Design

To characterise the expression of the DPP4 enzyme family in HCC, samples obtained during a previous study of HCC in mice were analysed [22]. Multiple insults including N-nitrosodiethylamine (DEN), thioacetamide (TAA) and a high-fat diet (HFD) were used to develop primary HCC in mice. Histopathology assessment was performed based on haematoxylin and eosin (H&E) stained sections. There are major similarities in histopathologic features in mouse, rat and human [23]. As such, the principles of human lesion identification have been applied to animal models. Focal areas of morphologically atypical hepatocytes in the liver are commonly defined as dysplastic foci (<1 mm) and dysplastic nodules (>1 mm). These dysplastic foci can be subclassified as displaying large-cell and small-cell change [24,25]. Dysplastic lesions can also be classified as either low-grade or high-grade dysplastic nodules [26]. Small-cell dysplasia (small-cell change), low-grade dysplastic nodules and high-grade dysplastic nodules are all considered to be precursors to HCC, either directly or indirectly, in a progression through stages [26]. The presence of stromal invasion is considered to be the hallmark feature that differentiates early HCC from dysplastic lesions [25]. Based on such criteria, lesions in DEN/TAA/HFD treated mice were identified by a certified pathologist as either dysplastic nodules or HCC. No control liver from untreated mice developed a dysplastic nodule or HCC. However, DEN/TAA- and DEN/TAA/HFD-treated mice had 3 ± 1.4 and 4 ± 0.7 dysplastic nodules (*p* < 0.01) and 0.2 ± 0.2 and 1.5 ± 0.5 HCC (*p* < 0.05), respectively [22].

For therapeutic evaluation, C57BL/6 mice were purchased from either Animal Resource Centre (Perth, WA, Australia) or Australian BioResources (Moss Vale, NSW, Australia). After one week of acclimation, mice were randomly distributed into control or treatment groups. The HCC murine model was similar to our established model [22] (Figure 4A). Briefly, N-nitrosodiethylamine (DEN; Sigma-Aldrich, St. Louis, MO, USA; catalogue number N0258-1G) was injected i.p. at 25 mg/kg body weight at 14 days of age. At 4 weeks of age, some mice drank thioacetamide (TAA) (Alfa Aesar, Shanghai, China; catalogue number A12926) at 300 mg/L acidified drinking water and ate a high-fat diet (HFD). At 16 weeks of age, ARI-4175 [19,20] at 6 mg/kg body weight was injected subcutaneously daily for 6 weeks. The dried 4175 was dissolved in 0.1 M HCl then diluted in saline. Control mice were injected with saline. Mice were co-housed, with ad libitum food and water, filtered air and a 12 h light/dark cycle. These experiments were approved and monitored by the Sydney Local Health District Animal Welfare Committee under ethics protocol 2017-030 (‘The biological roles of dipeptidyl peptidases’) and conducted in accordance with applicable laws and regulations.

### 2.2. Enzyme Assay

Enzyme assay was adapted from described methods [27] using the substrate H-Gly-Pro-pNA (Bachem, Bubenhof, Switzerland; catalogue number 4025614.0250). For each well, 50 μL substrate was added to 100 μg of protein lysate to a final concentration of 1 mM substrate in tris/EDTA (TE) buffer (pH 7.6) with 5% methanol. Absorbance was measured at 405 and 580 nm on a Polarstar plate reader (BMG Labtech, Ortenberg, Germany). DPP4 enzymatic activity was measured as a change in absorbance over 1 h at room temperature. To detect DPP8/9 enzymatic activity, 10 μM sitagliptin (a DPP4-selective inhibitor) was added to each well and change in absorbance was measured over 2 h at 37 °C [28].

### 2.3. Liver Lysate IL-1β Measurement

IL-1β in liver lysates was measured using an ELISA kit (R&D Systems, Inc., Minneapolis, MN, USA; catalogue number MLB00C) following the manufacturer’s instruction. A total of 115 μg of liver lysates was added per well for the measurement. Protein extracts of mouse livers were obtained as previously described [28]. Briefly, 10–20 mg of snap-frozen tissue was resuspended in ice-cold lysis buffer (50 mM Tris-HCl pH 7.6, 1 mM EDTA, 10% glycerol, 1% Triton X-100 and 1X Roche Complete Mini EDTA-free Protease Inhibitor Cocktail) at a ratio of 1:10 weight (µg)/volume (µL). The tissue was homogenised in a TissueLyser LT (Qiagen, Hilden, Germany, catalogue number 85600) using 7 mm stainless steel beads (Qiagen, catalogue number 69990). After homogenisation, samples were left on ice for a further 30 min and vortexed at 5 min intervals followed by centrifugation at 14,000 rpm for 20 min at 4 °C. The supernatant was extracted and quantified for protein concentration using the Micro BCA Protein Assay Kit (Thermo Fisher Scientific, Rockford, IL, USA, catalogue number 23235).

### 2.4. Western Blotting (WB)

Liver protein lysates were prepared as described above. NuPAGE Bis-Tris 4–12% gels (Invitrogen, Carlsbad, CA, USA; catalogue number NP0335) were used and procedures of Western blotting were performed as previously described [29]. Antibodies are listed in Table 1. The loading control was β-actin. Images were captured on a ChemiDocTM MP System (Bio-Rad Laboratories, Richmond, CA, USA). Densitometry was quantified using the Image Lab software version 5.2.1 (Bio-Rad Laboratories), with normalisation to β-actin.

### 2.5. Histology, Immunohistochemistry (IHC) and Image Analysis

Tissue samples were collected and fixed in 10% neutral buffered formalin and then processed by the Histopathology Core Facility in the University of Sydney. H&E staining, Picro-Sirius red staining and immunohistochemistry were completed as described previously [22,28]. Images were captured with a Leica DM6000B microscope (Wetzlar, Germany) and analysed with automation provided by Leica Application Suite X (LAS X) version 3.5.5.19976. Stain area thresholding was varied according to the isotype negative control immunostain. The immunostained area of CD8 or CD4 was measured using a threshold value and divided by total tissue area to calculate the proportion of positive staining on each section [22,30,31]. Histological quantification of lesions was performed as described [22].

### 2.6. Flow Cytometry

Liver tissue was collected and incubated in DMEM (Thermo Fisher Scientific; catalogue number 11965118), 10% FBS (GE Healthcare Life Sciences, Melbourne, VIC, Australia; catalogue number SH30084.03) and 1 mg/mL collagenase (Sigma-Aldrich; catalogue number C5138) for 1 h [32]. Afterward, leucocyte suspensions were obtained, and immunostaining was performed as described [22,33]. Cells were stained with LIVE/DEAD™ Fixable Blue Dead Cell Stain Kit (Thermo Fisher Scientific; catalogue number L34962, 1/200 dilution). Antibodies are listed in Table 2. Cells were analysed on a custom 10-laser LSR II (BD Biosciences, Franklin Lakes, NJ, USA). Flow cytometry data were analysed with FlowJo software version 9.9 (TreeStar, Ashland, OR, USA) [33].

### 2.7. Real-Time Quantitative PCR (qPCR)

Total liver RNA was extracted using the PureLink RNA Mini kit (Thermo Fisher; catalogue number 12183018A) and quantified using a Nanodrop spectrophotometer (Thermo Fisher Scientific; catalogue number ND-1000). The extracted RNA (1 μg) was reverse transcribed to cDNA using iScript™ cDNA synthesis kit (Bio-Rad Laboratories; catalogue number 1708891). The expression of genes of interest (Table 3) was measured, in 7-8 livers per group, by qPCR using the TaqMan™ Fast Advanced Master Mix (Thermo Fisher Scientific; catalogue number 4444963) and read using the LightCycler 480 Instrument II (Roche Life Science, Basel, Switzerland) and LightCycler 480 Software version 1.5 (Roche Life Science). Relative quantification used the Cτ (ΔΔCτ) method. Gene expressions were normalized to 2 endogenous controls, 18S and HPRT1.

### 2.8. Data Analysis

The Mann–Whitney U test was used for non-parametric datasets. The Kruskal–Wallis test with Dunn’s multiple comparison test was used for comparison among groups. Spearman’s correlation was performed for gene correlation. Data analysis and graph creation were performed in GraphPad Prism (GraphPad v. 9.9, San Diego, CA, USA). Significance was assigned to *p* value * *p* < 0.05, ** *p* < 0.01, *** *p* < 0.001 and **** *p* < 0.0001.

## 3. Results

### 3.1. Dipeptidyl Peptidase in Mouse HCC

In human HCC, the mRNA expression of the DPP4 gene family (*DPP9*, *DPP8, DPP4* and *FAP*) is greatly upregulated [9]. To investigate their roles in the pathogenesis of HCC, we characterized the DPP4 enzyme family in our validated HCC murine model that incorporates TAA, DEN and HFD [22].

**Figure 1:**
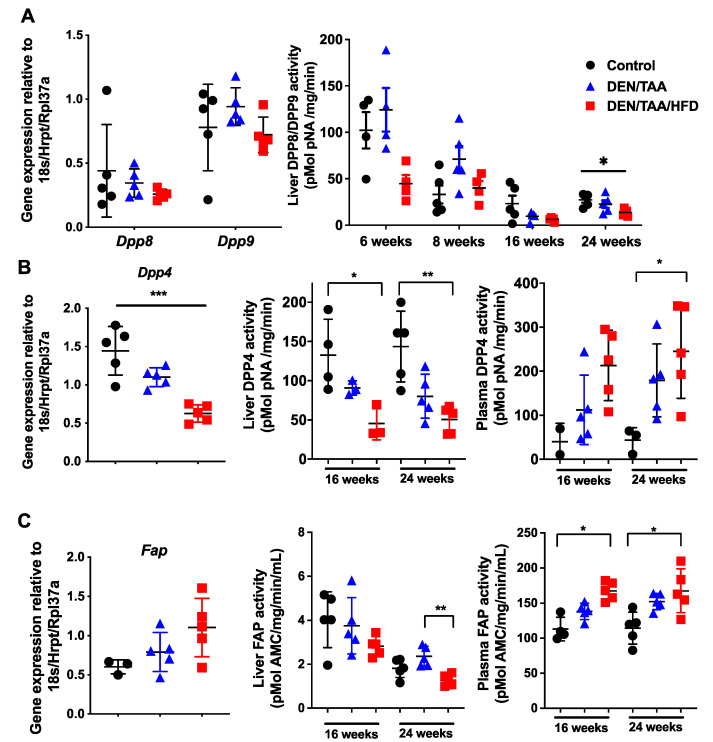
The DPP4 enzyme family in HCC. Normalised intrahepatic gene expression levels of *Dpp8* and *Dpp9* (**A**), *Dpp4* (**B**) and *Fap* (**C**). Enzymatic activity of DPP8 and DPP9 in liver (**A**). Enzymatic activities of DPP4 (**B**) and of FAP (**C**) in liver and plasma. Gene expression was at 24 weeks of age and normalised to *Hprt1/18S/Rpl37a*. Control was saline/chow. Mean ± SD, *n* = 3–6 per group. Statistical analysis used the Kruskal–Wallis test with Dunn’s multiple comparison test. Significance was ascribed by *p* value * *p* < 0.05, ** *p* < 0.01, *** *p* < 0.001.

We previously reported that intrahepatic *Dpp8* and *Dpp9* mRNA levels are upregulated in fibrotic mouse livers after 3 weeks of CCl_4_ treatment [34]. In the present study, no change in *Dpp8* or *Dpp9* whole-liver mRNA expression was observed among the control and two treatment groups (DEN/TAA and DEN/TAA/HFD groups) at 24 weeks of age (Figure 1A). Whole-liver DPP8/DPP9 enzyme activity in all groups, including the control group, decreased with age, irrespective of treatment. At 24 weeks of age, DEN/TAA/HFD treated livers had significantly reduced DPP8/DPP9 enzyme activity compared with control livers (Figure 1A). Measuring plasma DPP8/9 was not attempted because they are not detected in plasma [27], probably because these enzymes degrade rapidly outside of cells [35].

DPP4 is differentially expressed in chronic liver diseases and cancers in humans [36,37]. In our HCC model, intrahepatic mouse *Dpp4* mRNA expression significantly decreased at 24 weeks of age (Figure 1B). Whole-liver DPP4 enzyme activity decreased at both 16 and 24 weeks of age in treatment groups compared with the control group, whilst plasma DPP4 activity increased (Figure 1B). Increases in circulating DPP4 during chronic liver injury can be due to both cell death and the loss of hepatocyte polarity, which causes DPP4 to be shed from all cell surfaces rather than exclusively into the bile canaliculus [38,39]. Healthy, polarised hepatocytes express DPP4 only on the apical domain of the cell surface [39,40].

The whole-liver *Fap* mRNA level was not significantly changed in all groups at 24 weeks of age (Figure 1C). Similar to DPP4, liver FAP enzyme activity decreased whilst plasma FAP activity increased (Figure 1C), indicating that FAP may be shed from the liver to the plasma during liver injury, possibly from activated stellate cells and myofibroblasts.

**Figure 2:**
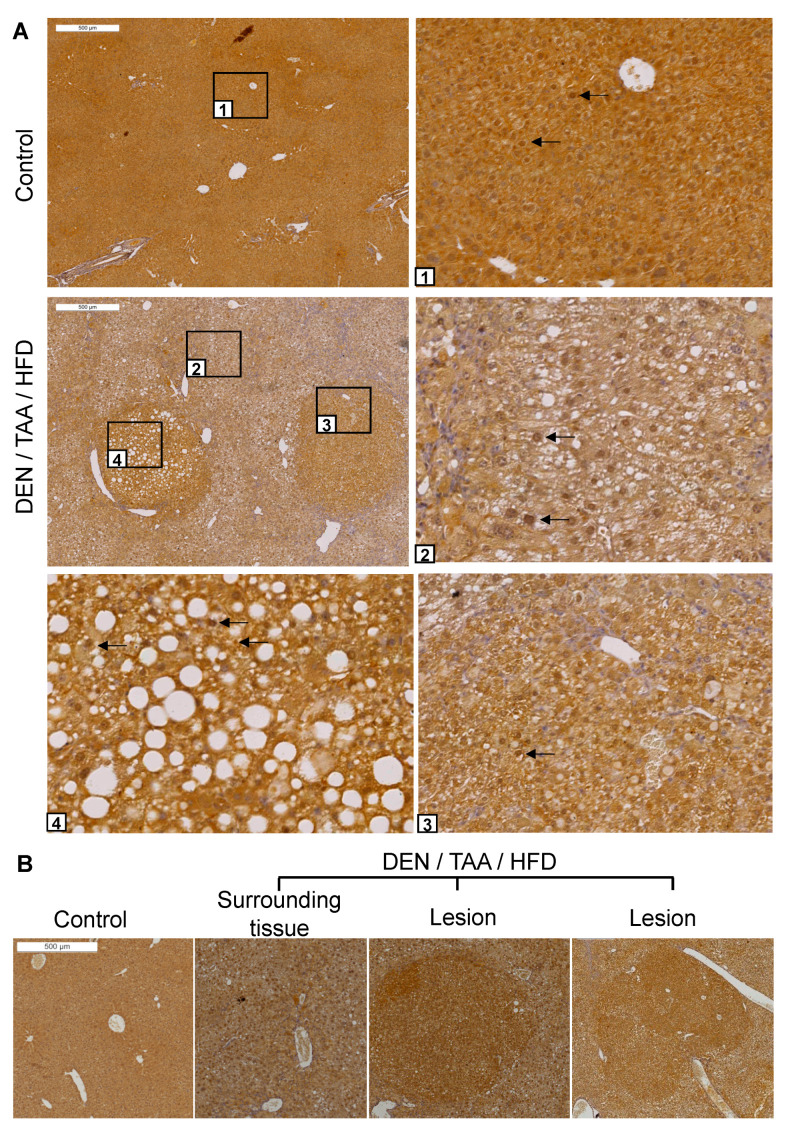
DPP9 immunostaining in liver sections. (**A**) Sections of saline/chow control and DEN/TAA/HFD-treated mice at 24 weeks of age were immunostained for DPP9 (brown). Boxed areas have magnified images of normal liver (1), diseased liver (2) and lesions (3,4). Black arrows point to some nuclei that were immunopositive for DPP9. (**B**) Representative image of DPP9 immunostaining. All 12 lesions of 4 mouse livers were intensely DPP9 immunopositive. Lesions were identified by encapsulation, increased steatosis and changes in hepatocyte morphology. Scale bars = 500 μm.

DPP9 protein abundance and location was also examined by immunohistochemistry. Intrahepatic DPP9 was ubiquitous and exhibited dense nuclear immunostaining in most cells (Figure 2A). Immunopositivity for DPP9 was greater in all lesions, including HCC, than in the surrounding liver (*n* = 4 mice; Figure 2A,B). This result is in agreement with with human HCC data that DPP9 is overexpressed in liver tumours more intensely than in normal tissue [9]. DPP9 may be selectively upregulated during carcinogenesis and might be a potential marker for precancerous lesions and tumours.

**Figure 3:**
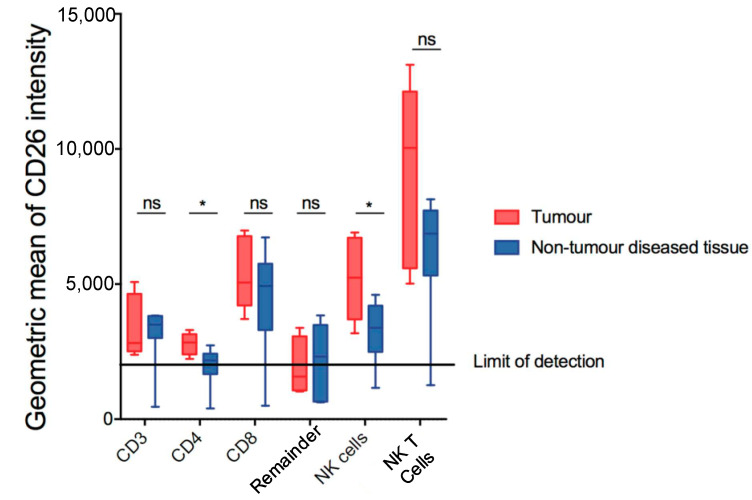
Measurement of DPP4 (CD26) in putative lymphoid subsets in DEN/TAA/HFD-treated livers at 32 weeks of age. CD26 geometric means of intensity of putative lymphoid subsets in tumour and non-tumour diseased tissue. Mean ± SD, *n* = 9 per group. Mann–Whitney U test, *p* value * *p* < 0.05. ns = not significant.

Given the role of DPP4 in immune regulation in HCC [14], lymphocyte populations in the DEN/TAA/HFD-treated mice were also assessed to determine if they retained expression of DPP4. The gating of putative T cells and natural killer cells is shown in Figure S1. Comparing tumour and non-tumour diseased tissue, we observed a modest increase in DPP4 expression by CD4^+^ T cells (CD45^+^ CD3^+^ CD4^+^ CD8^−^ NK1.1^−^) and NK cells (CD45^+^ NK1.1^+^ CD3^−^) (Figure 3). No difference in DPP4 expression was observed in the other populations examined.

### 3.2. ARI-4175 Decreased Liver Nodule Abundance and Increased Inflammatory Cell Infiltration in the Liver

**Figure 4:**
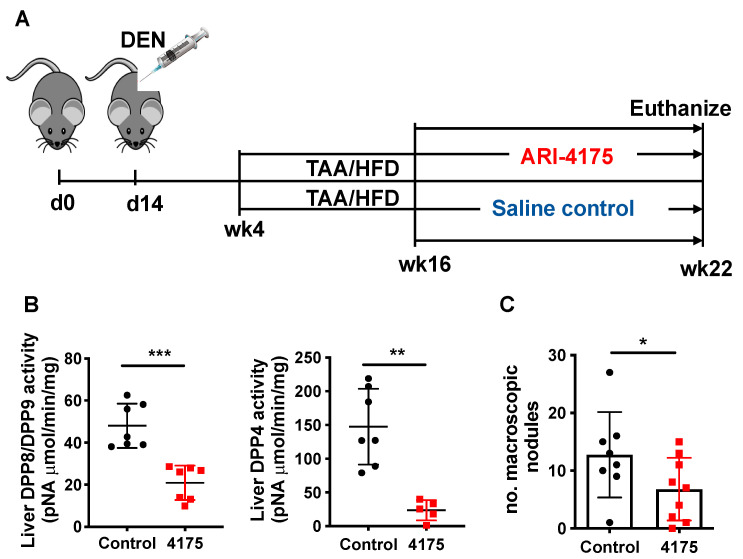
ARI-4175 in the DEN/TAA/HFD-induced HCC model. (**A**) ARI-4175 treatment regimen: N-nitrosodiethylamine (DEN; day 14)/thioacetamide (TAA; weeks 4–22)/high-fat diet (HFD; weeks 4–22). ARI-4175 (red square) or saline control (black circle) was injected during weeks 16–22. (**B**) DPP8/DPP9 and DPP4 enzymatic activities in liver at week 22. (**C**) Numbers of macroscopic nodules per liver in control and ARI-4175-treated mice at week 22. Mean ± SD, *n* = 7–9 per group. Mann–Whitney U test, *p* value * *p* < 0.05, ** *p* < 0.01, *** *p* < 0.001.

Inhibition of DPP4, DPP8, DPP9 and FAP by Val-boroPro has been shown to mediate regression in multiple tumour models, likely, at least in part, via the modulation of immune responses [10,12,21]. ARI-4175 is a second-generation pan-DPP inhibitor that enhances immunity in rhabdomyosarcoma [10] and lung tumours [13], but has not been assessed in HCC. We therefore evaluated the influence of ARI-4175 treatment on HCC occurrence and progression using our HCC model, with euthanasia at 22 weeks of age (Figure 4A). At harvest, no change in either body weight, liver weight or liver to body weight ratio was observed with treatment compared to the control (Figure S2), which is an indicator of drug safety. Enzyme assays of liver tissues showed successful inhibition of all four DPPs by ARI-4175 (Figure 4B and Figure S3). Notably, ARI-4175-treated mice had significantly fewer macroscopic liver nodules (Figure 4C), which likely indicates that ARI-4175 was able to reduce HCC occurrence during early- stage disease.

**Figure 5:**
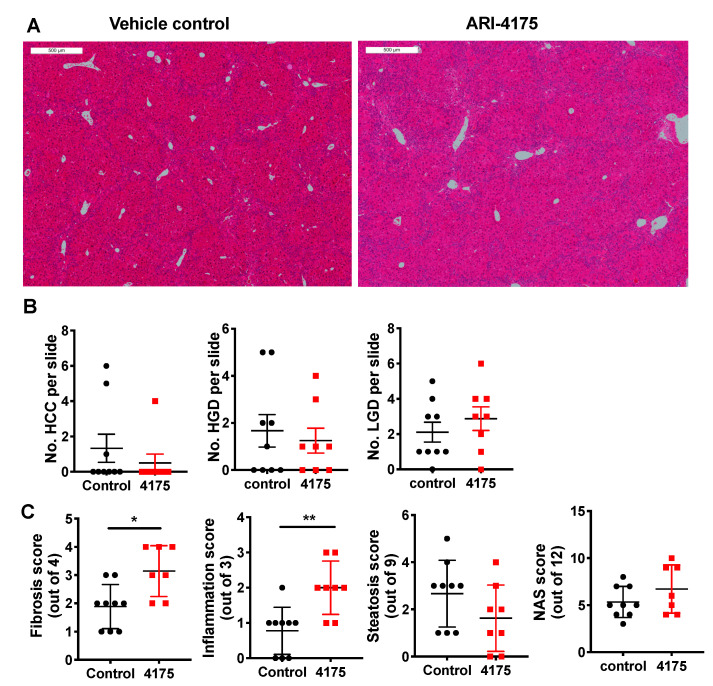
Histopathology following ARI-4175 treatment. (**A**) Representative images of H&E-stained liver sections at 22 weeks of age, scale bar = 500 μm. The histopathological scores obtained from this control mouse and this ARI-4175-treated mouse were: 1 and 0 for steatosis, 0 and 2 for inflammation, 3 and 2 for fibrosis and 4 and 4 for NAS, respectively. (**B**) Enumeration of HCC, high-grade dysplasia (HGD) and low-grade dysplasia (LGD) from H&E-stained liver sections. (**C**) Assessments of fibrosis, steatosis, inflammation and NAFLD activity score (NAS) from H&E-stained liver sections. ARI-4175 (red square), saline control (black circle). Mean ± SEM (B) or mean ± SD (**C**), *n* = 7–9 per group. Mann–Whitney U test, *p* values * *p* < 0.05, ** *p* < 0.01.

Liver histopathology (Figure 5A) was scored for low- and high-grade dysplastic lesions, and HCC. The principles for identification of lesions are described in the methods section. At the microscopic level, ARI-4175-treated mice had fewer high-grade dysplastic lesions and HCC, but those data lacked statistical significance, perhaps due to the small numbers of lesions (Figure 5B). The few lesions in a section of liver lobe compared to much more numerous nodules across the whole liver highlights a limitation of histopathology. Histological quantitation of fibrosis, steatosis, inflammatory cell aggregates and NAFLD activity score (NAS), performed as previously described [22], showed that ARI-4175 significantly increased fibrosis and inflammation, but not steatosis or NAS, compared to controls (Figure 5C).

**Figure 6:**
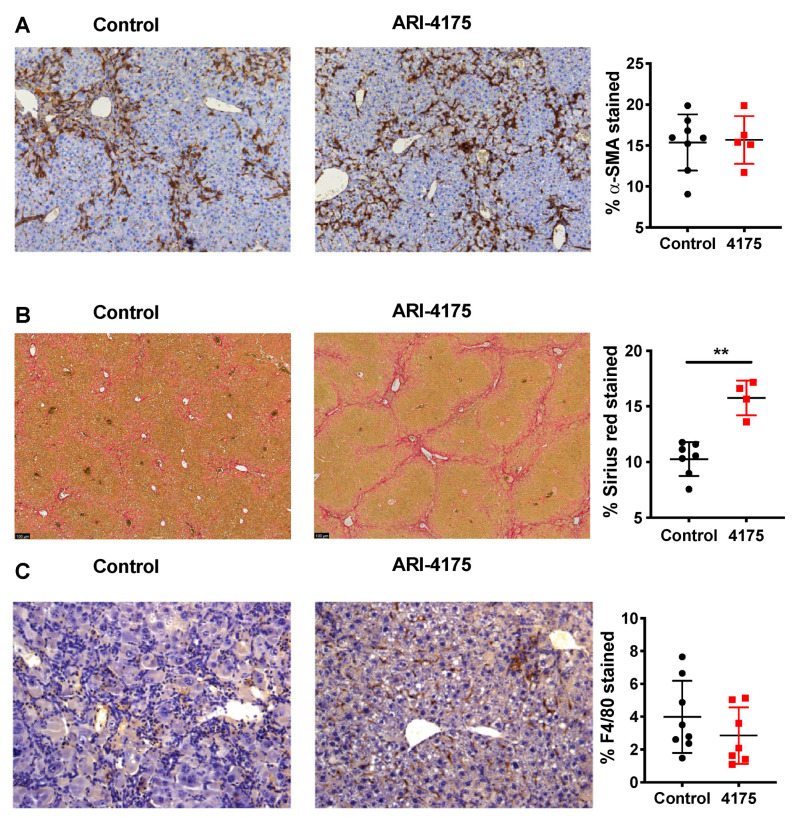
Assessment of activated hepatic stellate cells, fibrosis and macrophages following ARI-4175 treatment. Representative images of alpha-smooth muscle actin (α-SMA) immunostaining (**A**), Picro-Sirius Red stain (**B**) and F4/80 immunostaining (**C**). Hematoxylin counterstain (**A**,**C**). The stained area was measured and divided by total tissue area to calculate the proportion of positive staining. Mean ± SD, *n* = 4–8 per group. Mann–Whitney U test, *p* value ** *p* < 0.01.

Further assessment of activated hepatic stellate cells (aHSC) using alpha-smooth muscle actin (α-SMA) immunostaining showed no difference between control and treated livers (Figure 6A). However, there was significantly increased crosslinked collagen by Sirius red stain (Figure 6B), which aligned with the increased fibrosis scores that were obtained by assessing H&E-stained sections. Together, these data suggest a functional change in many aHSC, consisting of either increased extracellular matrix (ECM) production or decreased ECM digestion, caused by ARI-4175 treatment.

### 3.3. ARI-4175 Increased Intrahepatic CD8^+^ T Cell Density

**Figure 7:**
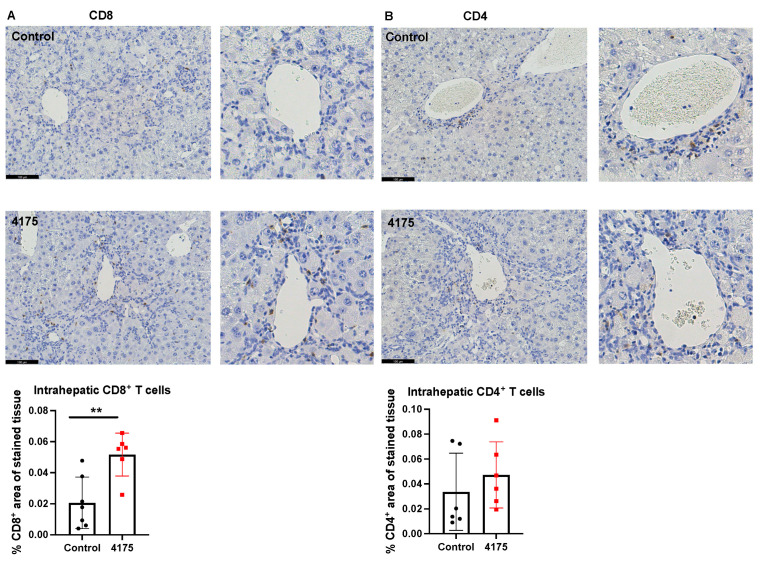
Quantitation of CD8^+^ and CD4^+^ T cells in livers. Representative images of CD8 (**A**) and CD4 (**B**) immunostains (brown stain) are presented. Higher magnification images of perivascular areas in the same field are on the right. The percentage area immunopositive for CD8 or CD4 in a liver section is graphed. The raw percentages of immunopositivity calculated from the two liver sections depicted here (control vs. 4175 treatment) are 0.0093% vs. 0.059% for CD8, 0.0013% vs. 0.013% for CD4. Mean ± SD, *n* = 6–7 per group. Mann–Whitney U test, *p* value ** *p* < 0.01. Scale bars = 100 μm.

To understand the underlying reason why ARI-4175 reduced the number of macroscopic nodules in HCC-bearing livers, the number and location of cytotoxic T cells were imaged and quantified based on the immunohistochemistry of CD8 in the liver. ARI-4175 enhanced intrahepatic CD8^+^ T cell recruitment (Figure 7A), consistent with the increased inflammation score by histology assessment. No difference in intrahepatic CD4^+^ T cells was observed (Figure 7B).

### 3.4. ARI-4175 Increased Intrahepatic Gene Expression of Macrophage Markers

**Figure 8:**
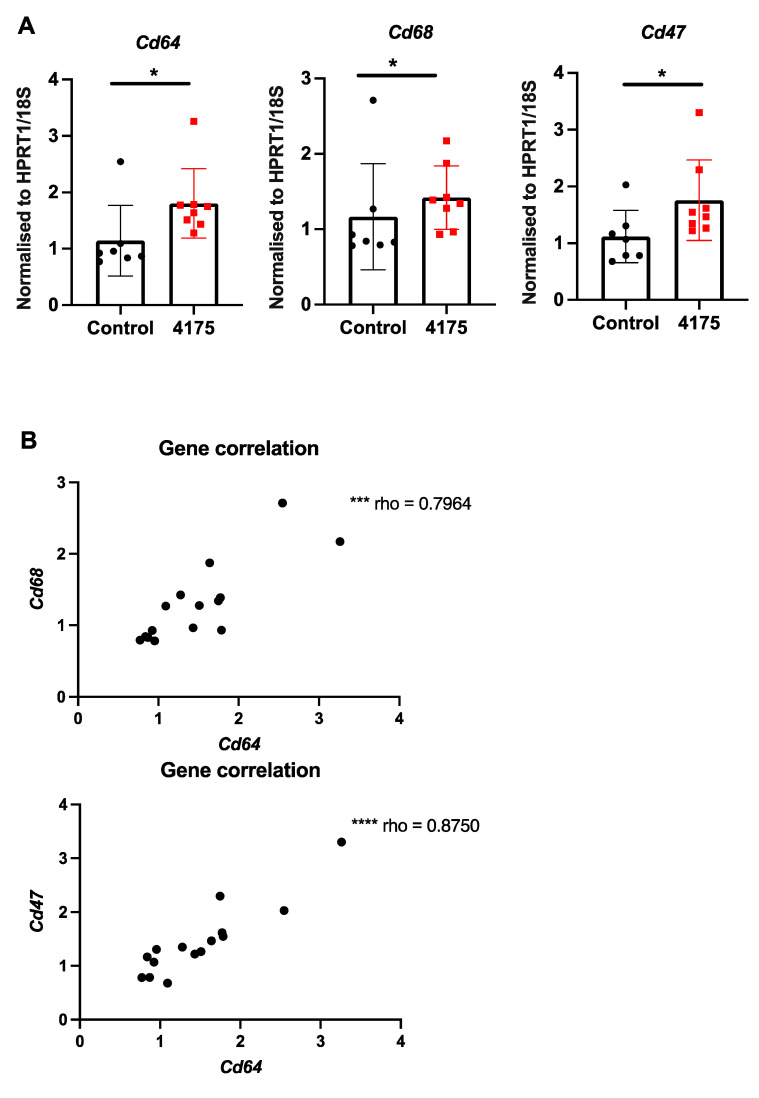
Expression of macrophage markers following ARI-4175 treatment. (**A**) Gene expression of *Cd64*, *Cd68* and *Cd47*, normalised to *Hprt1/18S*. (**B**) Correlations between gene expression of *Cd68* with *Cd64* and *Cd47* with *Cd64*. Mean ± SD, *n* = 7–8 per group. Mann–Whitney *U* test and Spearman correlation test, *p* values * *p* < 0.05, *** *p* < 0.001, **** *p* < 0.0001.

Since DPP9 has important roles in macrophage differentiation [41] and pyroptosis [19], we examined intrahepatic macrophages. Macrophage abundance by F4/80 immunostain did not differ between control and treated livers (Figure 6C). However, three macrophage-associated genes (*Cd64, Cd68, Cd47*) were significantly upregulated in ARI-4175-treated livers (Figure 8A), indicating a change in macrophage phenotype. The expression levels of *Cd64* were significantly correlated with both *Cd68* and *Cd47* (Figure 8B). Immunohistochemistry of CD47 and CD68 was performed. As expected, most cells were CD47^+^ but the cells immunostained most intensely formed a sinusoidal pattern, whereas CD68 immunopositivity was restricted to cells of macrophage/dendritic cell morphology. The CD47 and CD68 immunostained areas showed no difference between control and ARI-4175-treated livers (Figure S4), indicating that their mRNA and protein levels were discordant.

We also assessed intrahepatic gene expression associated with inflammation and fibrosis (Figure S5). A number of inflammatory genes were unaltered, *Col1a2* (collagen type I alpha 2 chain) was significantly increased, and *Gpc3* (glypican-3) was significantly decreased.

### 3.5. ARI-4175 Activation of A Caspase-1 Mediated Inflammasone Pathway

**Figure 9:**
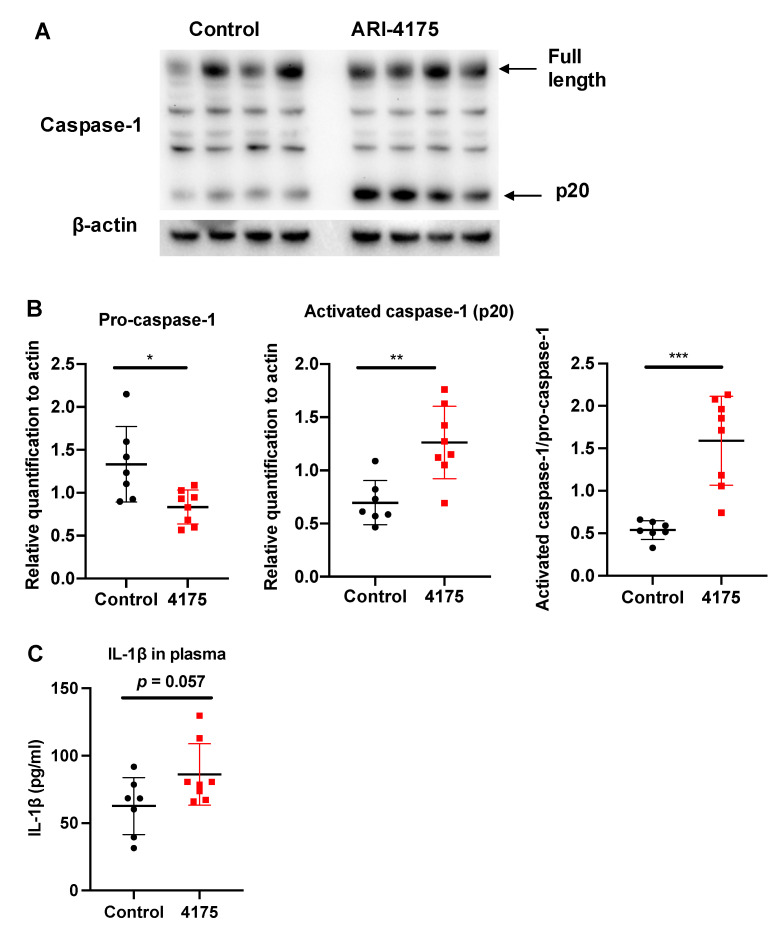
Assessment of inflammasome pathway components in liver following ARI-4175 treatment. Immunoblotting (**A**) and densitometry (**B**) for pro-caspase-1 and activated caspase-1 (p20). β-actin was the loading control. (**C**) IL-1β was measured by ELISA in liver lysates of control and ARI-4175 treated mice. Mean ± SD, *n* = 7–8 per group. Unpaired T test (for parametric data; **B**) and Mann–Whitney U test (for non-parametric data; **C**), *p* values * *p* < 0.05, ** *p* < 0.01, *** *p* < 0.001.

Each inflammasome is a multi-protein complex that mediates the activation of caspase-1, which promotes the secretion of the pro-inflammatory cytokines IL-1β and IL-18 [42]. DPP9 inhibition can activate the NLRP1b inflammasome, which in turn activates caspase-1 [17,18,20,43,44]. We wondered if ARI-4175 could affect the inflammasome pathway in HCC and then measured the intrahepatic levels of pro-caspase-1 (full length) and activated caspase-1 (cleaved form, called p20) by Western blotting. There was significantly more activated caspase-1 (p20) in ARI-4175-treated livers compared to control livers (Figure 9A). The ratio of activated caspase-1 to pro-caspase-1 was also significantly increased in ARI-4175-treated livers (Figure 9B). The level of IL-1β in the liver possibly has additional drivers, as it was not different in ARI-4175-treated livers (Figure 9C) and was not correlated with activated caspase-1 (Spearman’s rho = 0.164, *p* = 0.55). The mRNA levels of *pro-caspase-1*, *pro-caspase-3*, the inflammasomes *Nlrp1b* and *Nlrp3* and the cytokines *IL-1β* and *IL-18* showed no differences between treatment groups (Supplementary Figure S6). Autophagy markers were measured, because autophagy can induce inflammasome activation [45]. However, two autophagy markers, LC3B and Beclin-1, remained unchanged in ARI-4175-treated livers (Supplementary Figures S7 and S8).

## 4. Discussion

**Figure 10:**
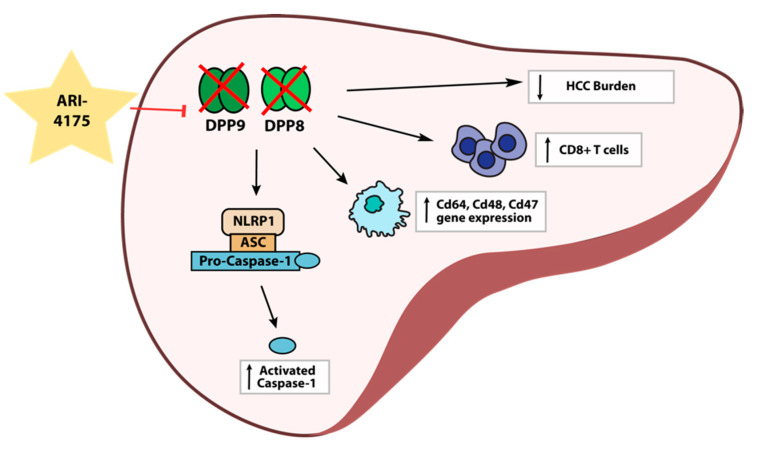
Working hypothesis of ARI-4175 treatment outcomes in the liver.

Despite the increased expression of DPP4 gene family members in human HCC, the role of these proteases in the development and progression of HCC is unclear. Here, we report for the first time on intrahepatic mRNA expression of the DPP4 gene family in HCC-bearing mice, along with measurements of their enzyme activities in both liver and plasma. We also demonstrate that the pan-DPP inhibitory compound ARI-4175 significantly reduced the number of macroscopic liver nodules while increasing the abundance of CD8^+^ T cells and of activated caspase-1 in a DEN/TAA/HFD-induced HCC model in mice. ARI-4175 increased intrahepatic inflammatory cell infiltration into the HCC-bearing liver, which corresponds with the increased CD8^+^ T cells and the caspase-1-mediated inflammasome activation, which is pro-inflammatory (Figure 10). Thus, this study provides promising data on the efficacy of ARI-4175 in the treatment of early-stage HCC.

Unlike DPPs in human liver with HCC [9] and mouse livers with CCl_4_-induced liver injury [34], mouse livers with DEN/TAA/HFD induced HCC showed decreased *Dpp4* mRNA levels, while *Dpp8, Dpp9* and *Fap* were unchanged. There are no data on the activity levels of these proteases in human HCC-bearing liver. From our mouse HCC data, these enzyme activities in the liver were downregulated over time, whilst DPP4 and FAP enzyme activities increased in the plasma. In human, increased plasma DPP4 and FAP enzyme activities have been reported in patients with NAFLD [46], liver fibrosis [47,48] and cirrhosis [49,50]. Therefore, in our DEN/TAA/HFD mice, the changes in enzyme activity in the liver and plasma were probably caused by cell death, loss of hepatocyte polarity, and fibroblast activation [39,51,52] rather than being related to tumour burden. In addition, we saw increased DPP4 (CD26) on CD4^+^ T cells and NK cells, which concurs with our previous observation that DPP4 gene knockout mouse livers had fewer intrahepatic CD4^+^ T cells in a liver fibrosis model [31]. Similarly, DPP4 inhibition can increase the abundance of activated NK cells (NKp46^+^) and CD3^+^ T cells in NASH-related HCC [14]. Thus, our data align with the increasing evidence of roles for DPP4 in regulating immune responses.

DPP9 enzyme activity is essential for early neonatal survival in mice. Mice with DPP9 enzyme ablation die shortly after birth [28,53]. Here, we show that intrahepatic DPP9 enzyme activity normally decreases with age in healthy mice. DPP9 is probably less important in later life, especially in immunity [54]. Mice with DEN/TAA/HFD treatment showed limited numbers of lesions immunopositive for AFP or GST-pi [22]. Nevertheless, 100% of lesions were DPP9-high, suggesting that DPP9 is potentially a candidate biomarker for neoplasm identification in this model, as well as indicating that tumours contain ample DPP9 for DPP9-targeted therapies.

HCC is associated with chronic liver disease, inflammation and fibrosis. Therapies must consider the underlying pathogenesis in the liver. DPP4 and FAP have established roles in metabolism, fatty liver, liver fibrosis and cirrhosis. Increasing evidence has linked DPP9 with inflammation and cytokine responses. A therapy targeting all of these proteases is therefore potentially a very effective immunotherapy. ARI-4175 is a second-generation pan-DPP inhibitor and, like Val-boroPro, has shown anti-cancer benefits [10,11]. Herein, we demonstrate that ARI-4175 significantly reduced the number of macroscopic nodules in the livers of DEN/TAA/HFD-treated mice compared with control mice (Figure 4C). The limited number of histologically confirmed HCC (Figure 5B) indicates that this model, at 22 weeks of age, recapitulates early tumour development. Inflammation is a known driver of fibrosis. The increased fibrosis and inflammation in the ARI-4175-treated mice is consistent with immune-based therapies, which are known to cause increased inflammation. However, the recent report that the DPP8/9 inhibitor TC-E5007 can lower collagen deposition in a renal fibrosis model suggests that outcomes of DPP8/9 inhibition might be context dependent [55].

We investigated the NLRP1 inflammasome pathway that DPP9 inhibition is able to activate [20]. The increased caspase-1 activation in ARI-4175-treated livers is the first in vivo evidence of DPP9 regulating intrahepatic inflammasomes. Our data further strengthen the importance of the regulation of the NLRP1 inflammasome by DPP9 [17,18]. Since we observed no change in the mRNA expression of inflammasome and related genes, this regulation is, as expected, only at the post-translational/protein level. Whether this inflammasome regulation occurred in hepatocytes, macrophages or lymphocytes in the liver is unknown. However, macrophages may have an important role in our model, as three macrophage-associated genes (*Cd64, Cd68, Cd47*) were significantly upregulated with ARI-4175 treatment, possibly indicating a functional change of those macrophages.

Inflammation can influence the host immune response to tumours and can be used in cancer immunotherapy [56]. Therapy-induced inflammation can enhance antigen presentation, leading to immune-mediated tumour eradication [57]. Adaptive immunity promotes immune surveillance to eradicate early HCC. The anti-tumorigenic role of T cells is facilitated through immune surveillance by CD4^+^ T cells and CD8^+^ T cells [58]. The stimulation of CD8^+^ cytotoxic T cells by immunotherapy has shown encouraging results in cancer therapy [59]. Here, in this study, the increased inflammation and CD8^+^ T cell abundance that we observed may be accompanied by an increased anti-tumour T-cell response. Since this mouse model recapitulates early tumour development, it was not feasible to quantify tumour-infiltrating lymphocytes. Moreover, ARI-4175 is a systemic therapy, so enumerating CD8^+^ and CD4^+^ T cells in the whole-liver section is more informative. The additional CD8^+^ T cells recruited to ARI-4175-treated livers might be a reason for the fewer macroscopic nodules that were observed in those mice. DPP4 can truncate CXCL10 to modify CXCL10–CXCR3 mediated influx of killer T cells in tumours [14,16]. Thus, DPP4 inhibition by ARI-4175 and its action on CXCL10–CXCR3 activation might facilitate the infiltration of CD8^+^ T cells.

In this study, FAP and DPP4 enzymatic activities were effectively inhibited with ARI-4175 treatment and increases in inflammation and fibrosis were observed. Several collagens are substrates of FAP [60,61], but pro-fibrotic TGFβ precursors are also FAP substrates [62], so the net outcome of FAP inhibition on fibrosis cannot be predicted. Another consideration is that specific DPP4 inhibition has been shown to decrease liver fibrosis [31] and slow HCC progression [63]. Glypican-3 (GPC3) binds to DPP4 [64] and is also a HCC marker. Whether the reduced level of Gpc3 relates to DPP4 levels or DPP4 inhibition is unknown. Although *Cxcl1*0 and *Cxcr3* remained unchanged at the mRNA level, their modifications by DPP4 at the protein level might occur and thus the CXCL10–CXCR3 might be an effector in our HCC murine model. In summary, several mechanisms for DPP4 and FAP involvement in the observed outcomes are known, but the dominant mechanism will be difficult to tease out.

These data are most consistent with a view that pan-DPP inhibitors might provide a useful treatment for early HCC with a focus on reducing tumour occurrence, rather than the treatment of established tumours. Since the pan-DPP inhibitor can harness the immune system to kill cancer cells, it could be used either as a stand-alone therapy, or in combination with other therapies that act via complementary mechanisms in HCC bearing livers. Increased inflammation, associated with fibrosis, might be a potential limitation if it persists after treatment. To manage the inflammation-related side effects, co-administration of a pan-DPP inhibitor with an inhibitor of cyclooxygenase (prostaglandin-endoperoxide synthase) has been found to retard PGE2-stimulated inflammation while at the same time allowing an enhanced T cell-mediated anti-tumoral immune response [65]. Therefore, such an approach may in the future be exploited to attenuate the pro-inflammatory and pro-fibrotic consequences of ARI-4175 administration in liver.

## 5. Conclusions

We provide proof-of-concept evidence that inhibiting the DPP4 enzyme family using ARI-4175 could reduce the number of macroscopic liver nodules, increase CD8^+^ T cell abundance and increase inflammasome activation during an early stage of development of HCC.

## Figures and Tables

**Table 1:** Antibodies for WB and IHC.

Antibody	Host	Supplier	Catalogue	Dilution	Application
Activated caspase-1 (P20)	Mouse	AdipoGen Life Sciences, San Diego, CA, USA	AG-20B-oo48-C100	1:1000	WB
Alpha-SMA	Rabbit	Abcam, Cambridge, UK	AB32575	1:100	IHC
Anti-mouse-HRP	Rabbit	Dako, Glostrup, Denmark	P0161	1:5000	WB
Anti-rabbit-HRP	Goat	Dako	P0448	1:5000	WB
Anti-rat-HRP	Goat	Invitrogen, Carlsbad, CA	31470	1:100	IHC
β-actin	Mouse	Abcam	Ab49900	1:50,000	WB
Beclin-1	Mouse	Genetex, Irvine, CA, USA	GTX34055	1:1500	WB
CD4	Rabbit	Cell Signaling Technology, Danvers, MA, USA	25229	1:50	IHC
CD8	Rabbit	Cell Signaling Technology	98941	1:150	IHC
Dipeptidyl peptidase 9	Rabbit	Abcam	Ab42080	1:100	IHC
F4/80	Rat	Dr Patrick Bertolino, Centenary Institute, Sydney, Australia	Hybridoma A3-1	neat	IHC
LC3B	Rabbit	Genetex	GTX127375	1:500–1:3000	IHC 1:500 WB 1:3000
Pro-caspase-1	Mouse	AdipoGen Life Sciences	AG-20B-0042-C100	1:1000	WB

**Table 2:** Antibodies for flow cytometry.

Antibody; Conjugate	Origin	Clone	Company	Catalogue	Dilution
CD45 BUV395	Rat (LOU) Anti-mouse IgG2b, κ	30-F11	BD Horizon	564279	1/100
CD3e BV421	Hamster Anti-mouse IgG1k	145-2C11	BD Horizon	562600	1/200
NK1.1 PE	Mouse Anti-mouse IgG2a, κ	PK136	BD Pharmingen	557391	1/100
CD26 PerCP-Cy5.5	Rat Anti-mouse IgG2a, κ	H194-112	eBioscience	45-0261-82	1/100
CD8a APC	Rat Anti-mouse IgG2a, κ	53–6.7	BD Pharmingen	553035	1/100
CD4 APC-Cy7	Rat Anti-mouse IgG2b, κ	GK1.5	BD Pharmingen	552051	1/100

**Table 3:** Probes used in qPCR.

Gene Name	Gene Symbol	Assay ID	Amplicon Length
Caspase-1	*Casp1*	Mm00438023_m1	99
Caspase-3	*Casp3*	Mm01195085_m1	70
CD47 antigen	*Cd47*	Mm00495011_m1	77
CD64 antigen	*Cd64*	Mm00438874_m1	58
CD68 antigen	*Cd68*	Mm00839636_g1	86
Chemokine (C-C motif) ligand 2	*Ccl2*	Mm99999056_m1	96
Chemokine (C-C motif) ligand 5	*Ccl5*	Mm01302427_m1	103
Chemokine (C-C motif) receptor 2	*Ccr2*	Mm99999051_gH	60
Chemokine (C-C motif) receptor 3	*Cxcr3*	Mm99999054_s1	57
Chemokine (C-X-C motif) ligand 10	*Cxcl10*	Mm00445235_m1	59
Chemokine (C-X3-X motif) receptor 1	*Cx3cr1*	Mm02620111_s1	107
Collagen, type I, alpha 2	*Col1a2*	Mm00483937_m1	77
Collagen, type III, alpha 1	*Col3a1*	Mm00802300_m1	88
Dipeptidyl peptidase 4	*Dpp4*	Mm00494538_m1	88
Dipeptidyl peptidase 8	*Dpp8*	Mm00547049_m1	95
Dipeptidyl peptidase 9	*Dpp9*	Mm00841122_m1	61
Eukaryotic 18S rRNA	*18S*	Hs99999901_s1	187
Fibroblast activation protein	*Fap*	Mm00484254_m1	107
Glypican 3	*Gpc3*	Mm00516722_m1	91
Hypoxanthine phosphoribosyltransferase 1	*Hprt1*	Mm00446968_m1	65
Interleukin 1 beta	*Il-1β*	Mm00434228_m1	90
Interleukin 18	*Il-18*	Mm00434226_m1	141
NLR family, pyrin domain containing 1B	*Nlrp1b*	Mm01241387_m1	68
NLR family, pyrin domain containing 3	*Nlrp3*	Mm00840904_m1	84
Nuclear factor of kappa light polypeptide gene enhancer in B cells inhibitor, alpha	*Nfkbia*	Mm00477798_m1	70
Nuclear factor of kappa light polypeptide gene enhancer in B cells inhibitor, beta	*Nfkbib*	Mm00456853_m1	64
Ribosomal protein L37a	*Rpl37a*	Mm01546394_s1	111
Tumour necrosis factor	*Tnf*	Mm00443258_m1	81

## Data Availability

The data presented in this study are available on request from the corresponding author.

## Supplementary Materials

The following are available online at https://www.mdpi.com/article/10.3390/cancers13215495/s1, Figure S1: Gating strategy for putative T cell and natural killer (NK) cell populations. Figure S2: Bodyweight and liver weight at 22 weeks of age. Figure S3: FAP enzyme activity in liver and plasma in control and ARI-4175 treated mice. Figure S4: Immunohistochemistry of CD47 and CD68 in liver in control and ARI-4175 treated mice. Figure S5: Intrahepatic gene expression levels of *Cxcl10, Cxcr3, Cxc3r1, Tnf-*α, *Nfkbia, Nfkbib, Ccl2. Ccr2, Ccl5, Dpp4, Fap, Dpp8, Dpp9, Col1a2, Col3a1* and *Gpc3* in control and ARI-4175-treated mice. Figure S6: Assessment of inflammasome pathway in control and ARI-4175-treated HCC livers at the mRNA level. Figures S7 and S8: Assessment of autophagy pathway components in control and ARI-4175-treated HCC livers.
