# Supplementary material for: Dipeptidyl Peptidase Inhibition Enhances CD8 T Cell Recruitment and Activates Intrahepatic Inflammasome in a Murine Model of Hepatocellular Carcinoma
Source: Cancers (Basel). 2021 Nov 1;13(21):5495. doi: 10.3390/cancers13215495 (PMC8583374; doi:10.3390/cancers13215495)
Supplement: Supplementary file 1 [file cancers-13-05495-s001.zip › cancers-1357026-supplementary.pdf]

cancers

Dipeptidyl Peptidase Inhibition Enhances CD8 T Cell Recruitment and Activates Intrahepatic Inflammasome in a Murine Model of Hepatocellular Carcinoma

James M. Henderson [1,2], Michelle S. W. Xiang [1], Jiali Carrie Huang [1], Stefanie Wetzel [1], Linxuan Jiang [1], Jack H. Lai [3], Wengen Wu [3], James G. Kench [4], William W. Bachovchin [3], Ben Roediger [1], Geoffrey W. McCaughan [1,5], Hui Emma Zhang [1,*] and Mark D. Gorrell [1,*]

1 Centenary Institute, Faculty of Medicine and Health, The University of Sydney, Camperdown, NSW 2006, Australia; jhen7621@gmail.com (J.M.H.); m.xiang@centenary.org.au (M.S.W.X.); jhua9515@uni.sydney.edu.au (J.C.H.); christian.stefanie@yahoo.com (S.W.); ljia7302@uni.sydney.edu.au (L.J.); ben.roediger@novarits.com (B.R.); g.mccaughan@centenary.org.au (G.W.M.)

2 Institute for Cardiovascular Prevention, Ludwig-Maximillians-Universität, D-80336 Munich, Germany

3 Sackler School of Graduate Biomedical Science, Tufts University, Boston, MA 02111, USA; jack.lai@tufts.edu (J.H.L.); WenGen.Wu@tufts.edu (W.W.); william.bachovchin@tufts.edu (W.W.B.)

4 Tissue Pathology & Diagnostic Oncology, NSW Health Pathology, Royal Prince Alfred Hospital, Camperdown, NSW 2050, Australia; James.Kench@sydney.edu.au

5 A.W. Morrow Gastroenterology and Liver Centre, Royal Prince Alfred Hospital, Camperdown, NSW 2050, Australia

* Correspondence: e.zhang@centenary.org.au (H.E.Z.); m.gorrell@centenary.org.au (M.D.G.); Tel.: +61-2-95656156 (M.D.G.)

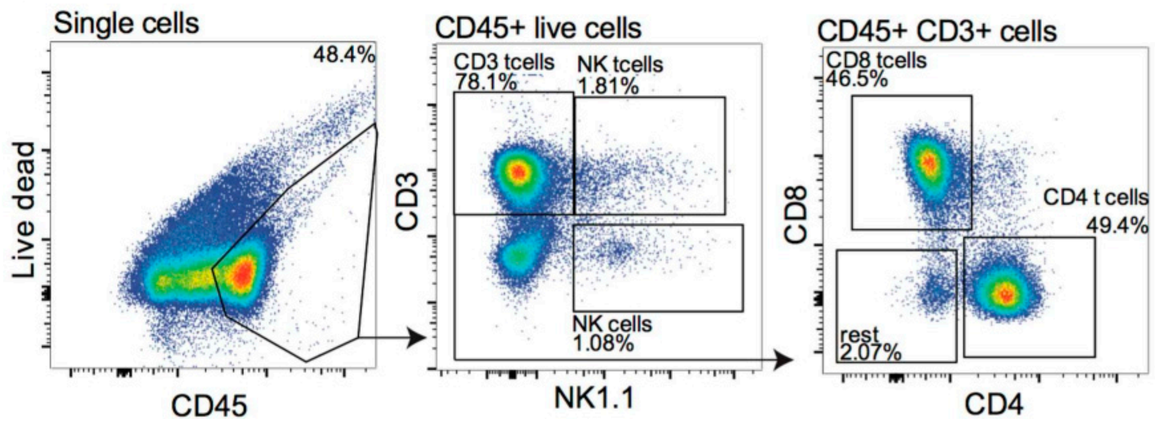

Supplementary Figure S1. Gating strategy for putative T cell and natural killer (NK) cell populations. Number adjacent to each outlined area is positive cells as percentage of each parent population.

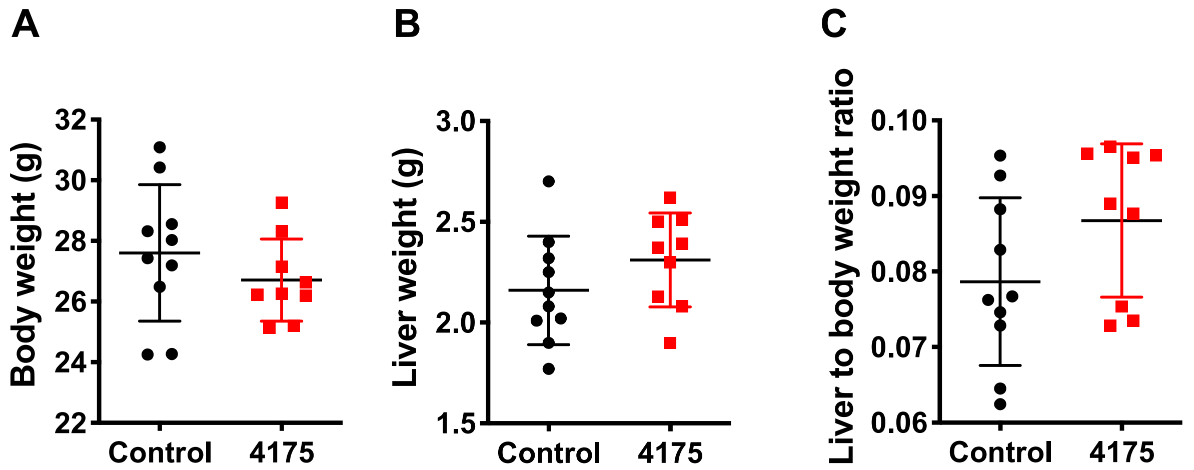

Supplementary Figure S2. Bodyweight and liver weight at 22 weeks of age. Mouse body weight (**A**), mouse liver weight (**B**), and liver to body weight ratio (**C**). Mean ± SD, n = 9-10 per group. Mann-Whitney U test.

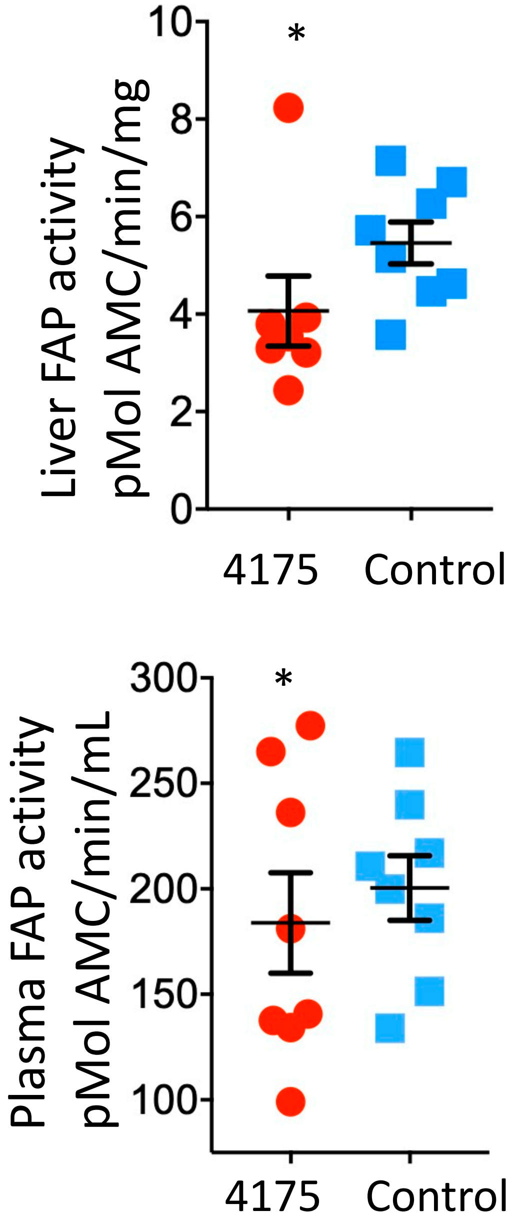

Supplementary Figure S3. FAP enzymatic activities in liver and blood plasma at 22 weeks of age. Mean ± SD, n = 9-10 per group. Mann-Whitney U test, * $p < 0.05$.

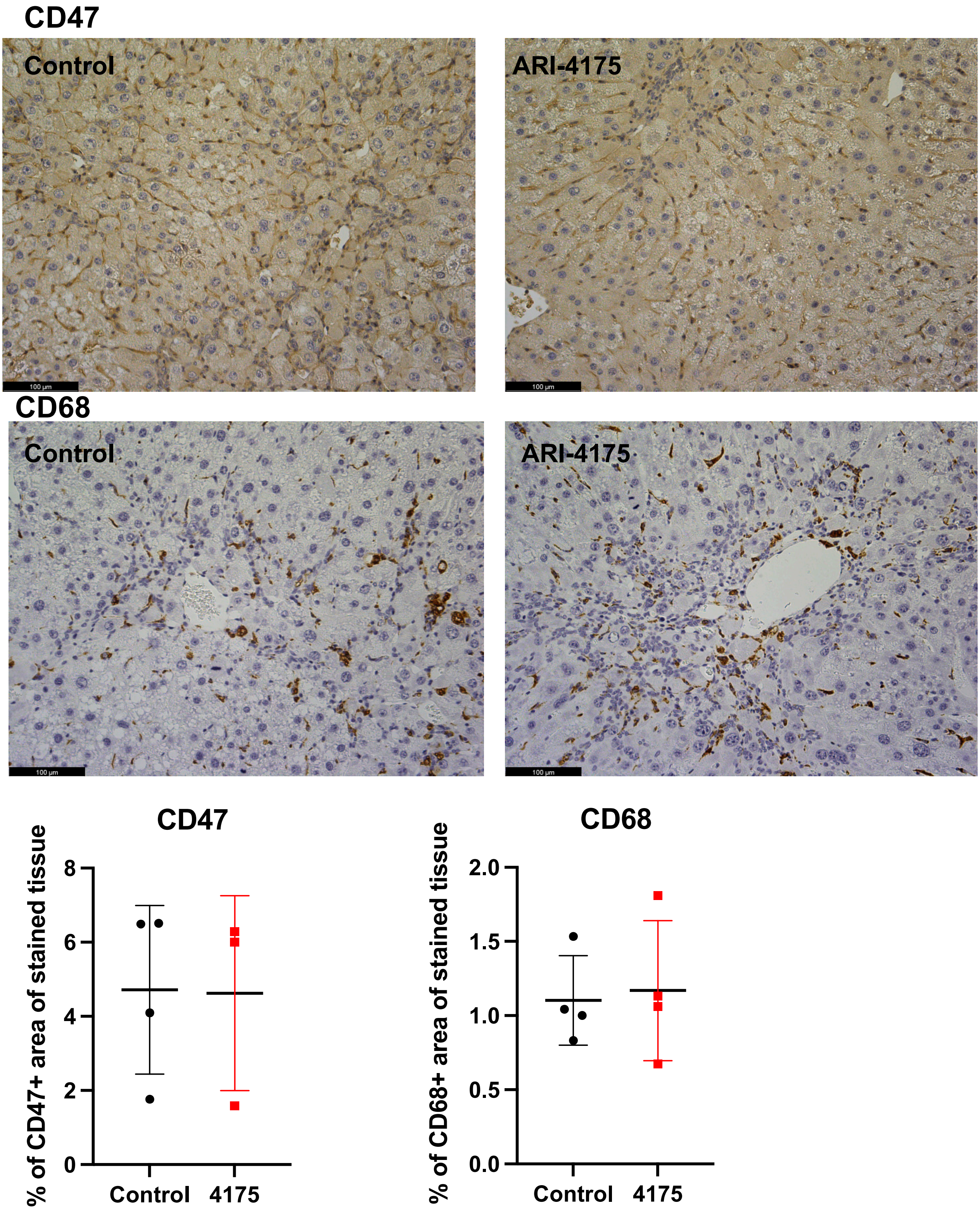

Supplementary Figure S4. Assessment of CD47 and CD68 immunostains following ARI-4175 treatment. Most cells were $CD47^{+}$ but a sinusoidal pattern was evident. CD68 immunopositivity appeared restricted to cells of macrophage/ dendritic cell morphology. Representative immunohistochemistry images are shown. The stained area was measured and divided by total tissue area to calculate proportion of positive staining. Mean ± SD, n = 3-4 per group.

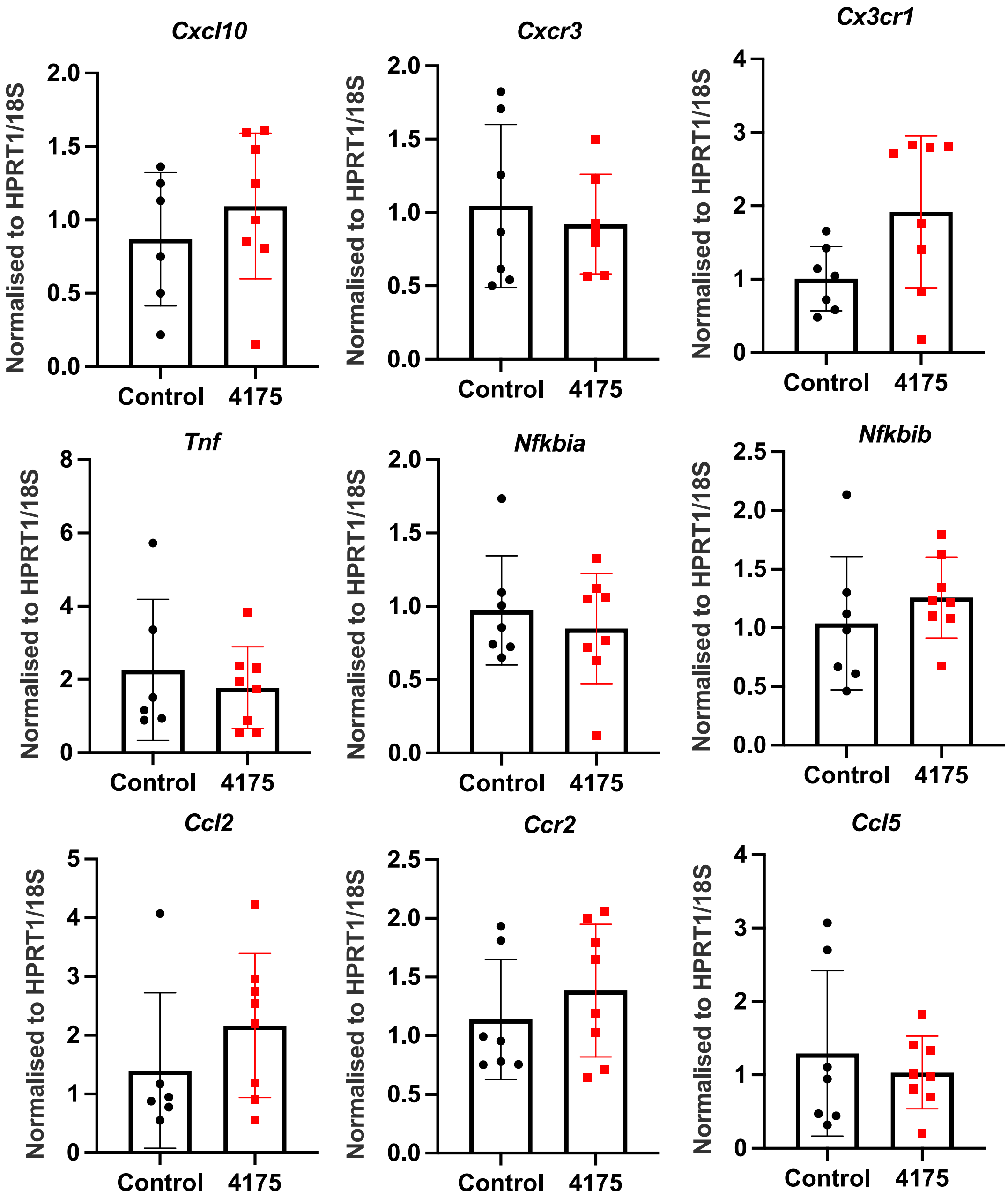

Cxcl10
Cxcr3
Cx3cr1
Tnf
Nfkbia
Nfkbib
Ccl2
Ccr2
Ccl5
Normalised to HPRT1/18S
Control
4175

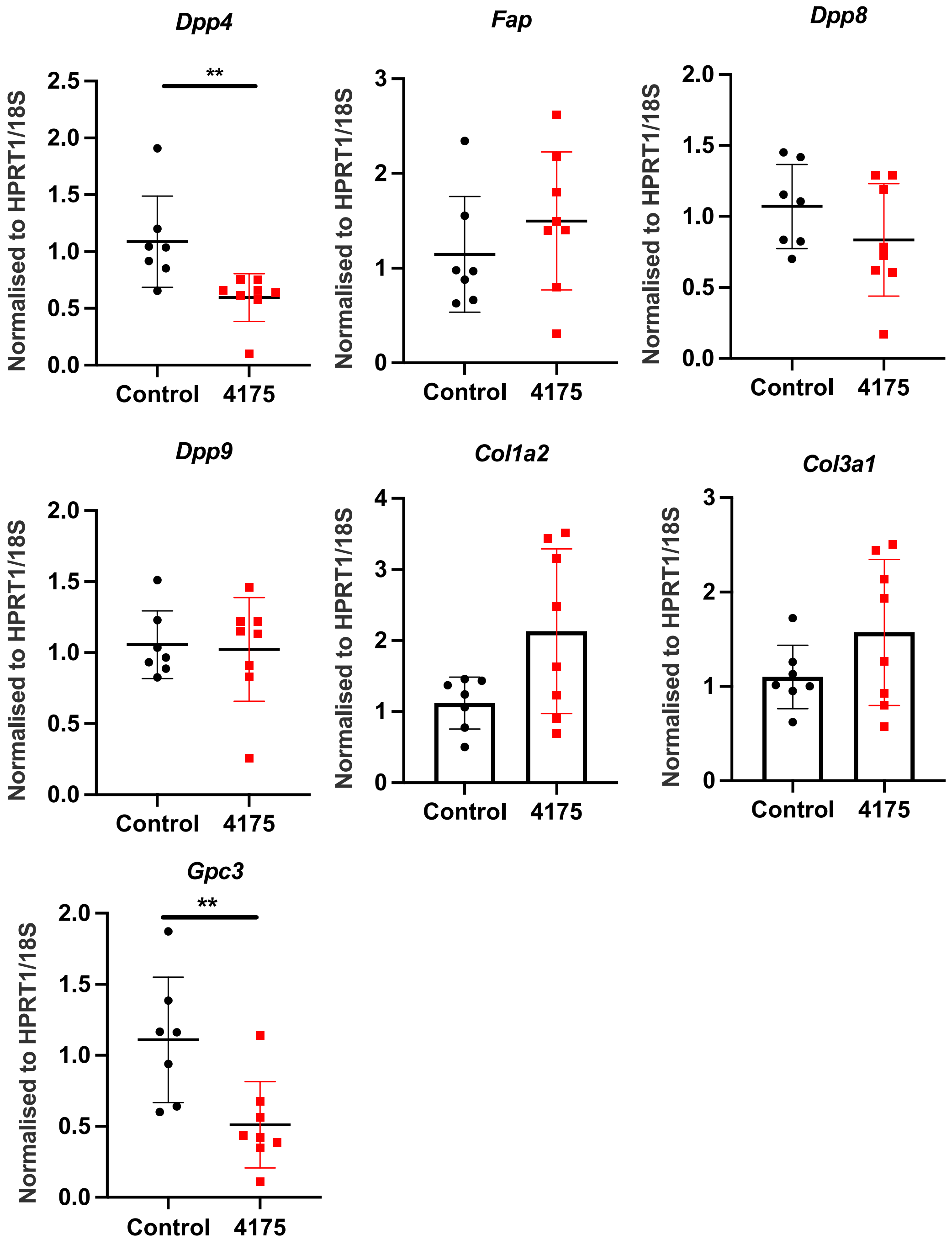

Supplementary Figure S5. Intrahepatic gene expression levels of *Cxcl10, Cxcr3, Cxc3r1, Tnf-α, Nfkbia, Nfkbib, Ccl2. Ccr2, Ccl5, Dpp4, Fap, Dpp8, Dpp9, Col1a2, Col3a1* and *Gpc3* in control and ARI-4175 treated mice. Gene expression was normalised to *Hprt1/18S*. Mean ± SD, n = 7-8 per group. Mann-Whitney U test, *p* values * < 0.05, ** < 0.01.

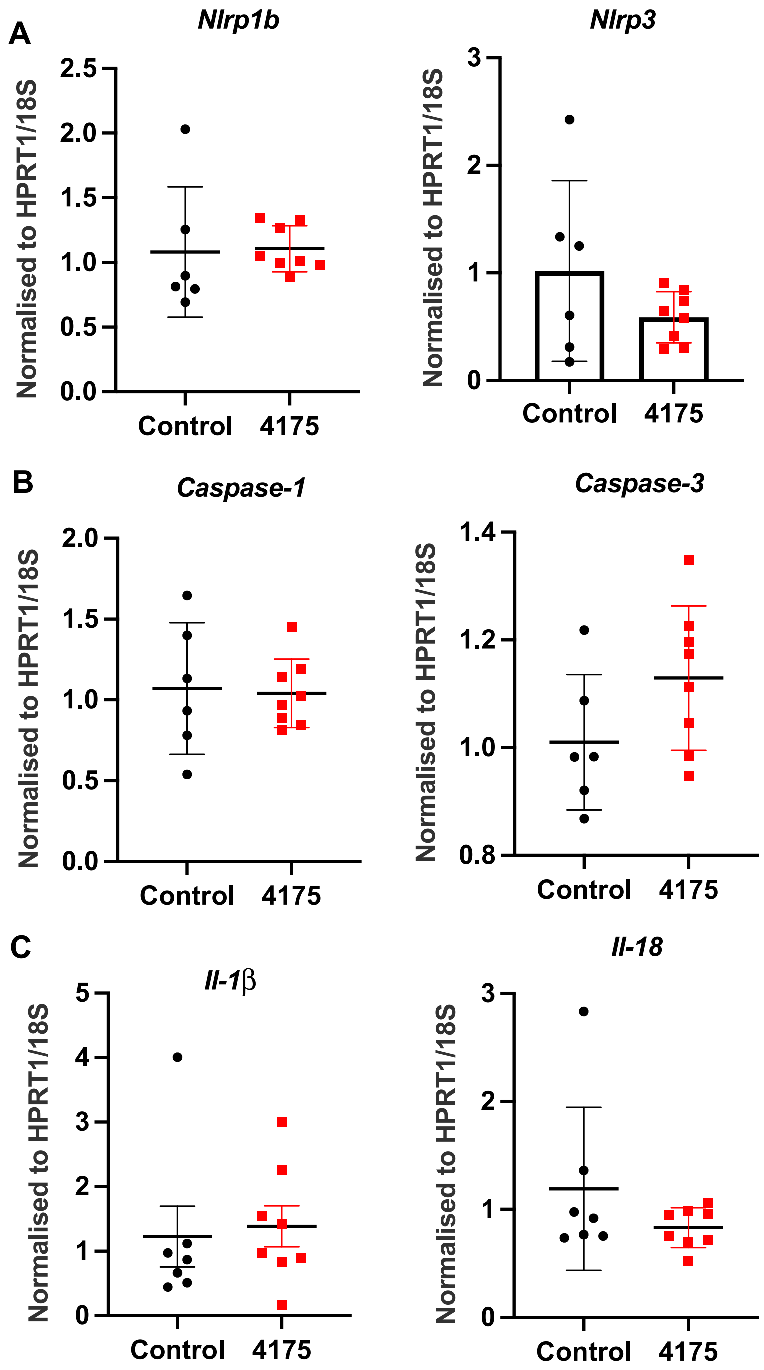

Supplementary Figure S6. Assessment of inflammasome pathway in control and ARI-4175 treated HCC livers at the mRNA level. The gene expression levels of *Nlrp1b*, *Nlrp3* **(A)**, *pro-caspase-1*, *pro-caspase-3* **(B)**, and *Il-1β*, *Il-18* **(C)** were normalised to *Hprt1/18S*. Mean ± SD, n = 7-8 per treatment group. Mann-Whitney U test.

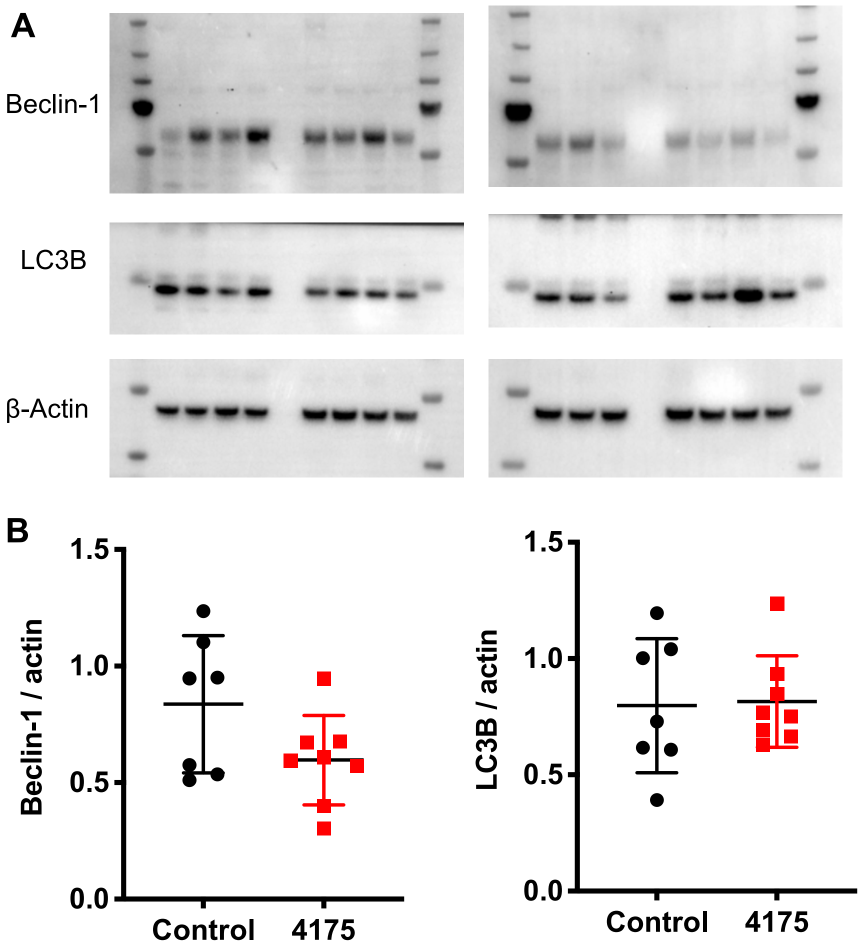

Supplementary Figure S7. Assessment of autophagy pathway components in control and ARI-4175 treated HCC livers. Immunoblotting (**A**) and densitometry (**B**) for autophagy markers Beclin-1 and LC3B were quantified in liver lysates. β-actin was the loading control. Mean ± SD, n = 7-8 per group. Mann-Whitney U test.

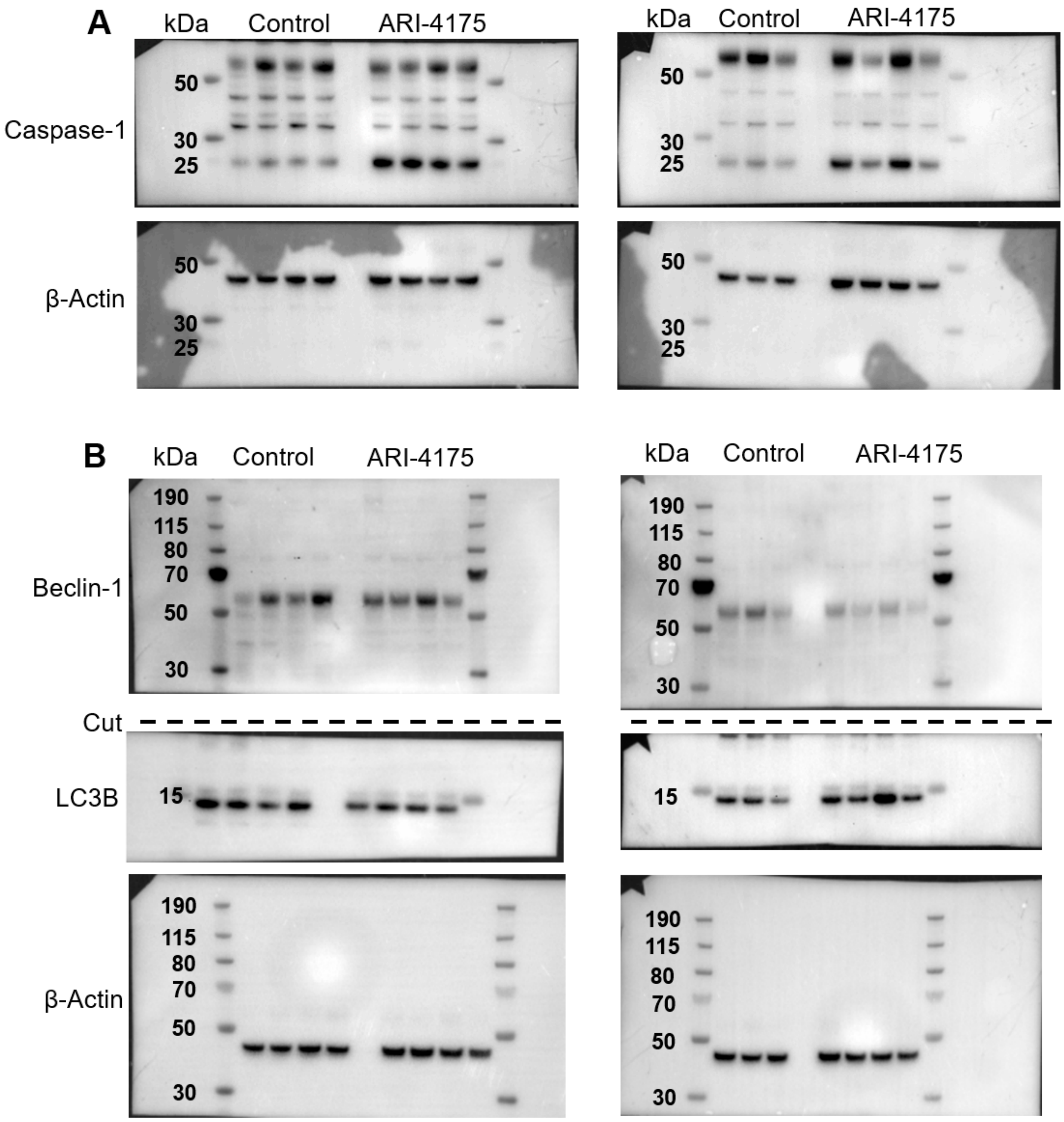

Supplementary Figure S8. Full-length immunoblots for (**A**) Caspase-1 and (**B**) Autophagy markers Beclin-1 and LC3B in control and ARI-4175 treated HCC liver lysates. β-actin was the loading control. n = 7 for control and n = 8 for ARI-4175 treatment.
